# Nocturnal gastro-oesophageal reflux and pulmonary abnormalities on chest CT in a general population: the Swedish CArdioPulmonary BioImage Study

**DOI:** 10.1136/thorax-2024-222570

**Published:** 2025-08-10

**Authors:** Össur Ingi Emilsson, Andrei Malinovschi, Åse Johnsson, Mirjam Ljunggren, Anders Blomberg, Ida Pesonen, Magnus Sköld, Zainab Ahmadi, Anna Moberg, Tomas Hansen

**Affiliations:** 1 Department of Medical Sciences, Respiratory, Allergy and Sleep Research, Uppsala University, Uppsala, Sweden; 2 Faculty of Medicine, University of Iceland, Reykjavik, Iceland; 3 Medical Sciences, Clinical Physiology, Uppsala University, Uppsala, Sweden; 4 Department of Radiology, Sahlgrenska University Hospital, Goteborg, Sweden; 5 Department of Radiology, Institute of Clinical Sciences, Sahlgrenska Academy, University of Gothenburg, Gothenburg, Sweden; 6 Public Health and Clinical Medicine, Umeå University, Umea, Sweden; 7 Department of Medicine, Respiratory Medicine Unit, Center for Molecular Medicine, Karolinska Institutet, Stockholm, Sweden; 8 Lung Allergy Clinic, Karolinska Universitetssjukhuset, Stockholm, Sweden; 9 Department of Clinical Sciences, Respiratory Medicine, Allergology, and Palliative Medicine, Lund University, Lund, Sweden; 10 Centre of Medical Image Science and Visualization, Linköping University, Linkoping, Sweden; 11 Department of Health, Medicine and Caring Sciences, Linköping University, Linkoping, Sweden; 12 Department of Surgical Sciences, Radiology, Uppsala University, Uppsala, Sweden

**Keywords:** Interstitial Fibrosis, Bronchiectasis, Imaging/CT MRI etc

## Abstract

**Background:**

Nocturnal gastro-oesophageal reflux (nGER) is common in people with respiratory diseases, but its association with pulmonary abnormalities is not known.

**Aim:**

Investigate the association between nGER and pulmonary abnormalities on chest CT in an adult general population.

**Methods:**

In total, 28 846 individuals from the general population aged 50–64 years completed questionnaires and underwent chest CT, in the Swedish CArdioPulmonary BioImage Study (www.scapis.org). Participants with nGER symptoms on ≥1 night per week were defined as having nGER. Chest CT was evaluated for bronchial wall thickening, bronchiectasis, reticular abnormalities, honeycombing, cysts and ground glass opacities. Ever-smoking, current asthma, inflammatory bowel disease and autoimmune disease were defined as risk factors for pulmonary abnormalities. Analyses were adjusted for sex, age, body mass index, education level and study centre.

**Results:**

The prevalence of nGER was 9.4%. Among participants with risk factors for pulmonary abnormalities (n=4004), having nGER was positively associated with bronchial wall thickening (adjusted OR (aOR) (95% CI): 1.25 (1.07 to 1.48)) and reticular abnormalities (aOR (95% CI): 1.51 (1.04 to 2.17)), but negatively associated with cysts (aOR (95% CI): 0.68 (0.48 to 0.97)). Among participants without risk factors for CT abnormalities (n=2555), nGER did not relate with pulmonary abnormalities.

**Conclusions:**

In a middle-aged general population, nGER was not associated with pulmonary abnormalities on chest CT. However, in the presence of other risk factors for pulmonary abnormalities, nGER was associated with bronchial wall thickening and reticular abnormalities. Persons with nGER and risk factors for pulmonary abnormalities should, therefore, be evaluated for respiratory disease and treated appropriately.

**WHAT IS ALREADY KNOWN ON THIS TOPIC**

- Gastro-oesophageal reflux is often considered a risk factor for interstitial lung disease and airways disease. However, good evidence is lacking, especially for early stages of disease.

**WHAT THIS STUDY ADDS**

- In the general population, nocturnal gastroesophageal reflux (nGER) does not independently associate with pulmonary abnormalities on chest CT. However, among individuals with other risk factors for developing pulmonary abnormalities (smoking history, asthma, inflammatory bowel disease or systemic autoimmune disease), a significant although relatively weak association was found between nGER and bronchial wall thickening and reticular abnormalities.

**HOW THIS STUDY MIGHT AFFECT RESEARCH, PRACTICE OR POLICY**

- These results show that in an otherwise healthy general population, nGER should not be considered a risk factor for developing pulmonary disease. On the other hand, when combined with other risk factors predisposing to pulmonary abnormalities, people with nGER may be at increased risk of developing pulmonary disease.

## Introduction

Nocturnal gastro-oesophageal reflux (nGER) is a common condition, characterised by night-time reflux of gastric content in the oesophagus, oral cavity or lungs. The main symptoms are heartburn and acid regurgitation.^1 2^ nGER has been suggested to be a risk factor for the development or worsening of diseases such as interstitial lung disease (ILD), chronic obstructive pulmonary disease (COPD), bronchiectasis, chronic cough and asthma.^13^,^5^ This association has been reported in a number of epidemiological studies, but the underlying mechanisms are not fully understood. Several hypotheses have been proposed, including reflux of gastric liquid content to the airways (ie, microaspiration), and inhalation of gastroenteric gases.^6^ This has led to antacid therapies being frequently prescribed for patients with respiratory disease, for example, in ILD, even though patients often have concerns about potential side effects.^7^ However, recent studies have begun to question the relevance of nGER in ILD, for example, as it does not seem to impact hospitalisations or mortality.^8^ Also, treating patients with idiopathic pulmonary fibrosis (IPF) with antacid therapies has not been shown to impact the progress of IPF.^9^ Antacid treatments also have a limited effect on chronic cough. It is, therefore, of high importance to better understand this potential association to inform treatment decisions.

Many respiratory conditions show radiological lung abnormalities on CT. In ILD, reticular abnormalities, ground-glass opacities, traction bronchiectasis and honeycombing are common findings.^10^ Bronchiectasis, a heterogeneous and multifactorial condition,^11^ is identified on chest CT by a non-tapering appearance of a bronchus within one centimetre from the pleura, bronchial wall thickening and a wider bronchial lumen than an adjacent pulmonary artery.^12^ Asthma can be associated with mosaic attenuation, bronchiectasis and bronchial wall thickening.^13^ Given that nGER is a proposed risk factor for these diseases, it is plausible that nGER should be associated with lung abnormalities on chest CT, but data on this possible association are scarce. It is, therefore, unclear if the presence of nGER should raise suspicion of structural abnormalities in general, motivating radiological evaluation.

In some cases, pulmonary abnormalities involving the lung interstitium are called interstitial lung abnormalities (ILA), which is a radiological definition of incidentally found patterns affecting >5% of any lung zone in an individual without a clinical suspicion of ILD. A recent publication on ILA in the same general population sample as studied here found no association between nGER and ILA.^14^ However, that analysis did not include bronchiectasis or bronchial wall thickening without other ILA-related abnormalities, nor did it specifically examine the severity of nGER symptoms or whether other risk factors could influence a potential association with lung abnormalities on chest CT. Therefore, that analysis could not draw conclusions about the potential association between nGER and pulmonary abnormalities. A more directed analysis including a broader spectrum of pulmonary abnormalities, with a focus on nGER and nGER severity is therefore warranted.

The aim of this study was to investigate if nGER associates with interstitial and bronchial lung abnormalities on chest CT in a middle-aged general population, with or without known risk factors for lung abnormalities. This was done within a cross-sectional general population study including chest CT scans on roughly 30 000 middle-aged adults.

## Methods

### Study population

The Swedish CArdioPulmonary Bioimage Study (SCAPIS) is a large, multicentre, observational study, carried out in 2013–2018. The total SCAPIS cohort consists of 30 154 participants aged 50–64 years randomly selected from the general population. The participants were randomly recruited from the general population of six regions in Sweden (Uppsala, Umeå, Linköping, Malmö/Lund, Gothenburg and Stockholm); on their visit, they answered detailed questionnaires, performed spirometry, gave blood samples and underwent a chest CT scan, usually within 1 week. Of these, 28 846 (96%) had answered questions on nGER and were therefore included in the present study. A detailed description of the objectives and design of SCAPIS has previously been published.^15^ The SCAPIS cohort has been found to represent well the Swedish general population of the same age.^16^

### Nocturnal gastro-oesophageal reflux

nGER was defined by participants’ self-reported frequency of heartburn or regurgitation after going to bed, with slight modification.^2^ Participants were defined to be ‘without nGER’ if they never/almost never had reflux symptoms. Participants with symptoms less than once a week were defined as having ‘possible nGER’. Participants with symptoms at least one night per week were defined as ‘with nGER’.

### Chest CT imaging

The chest of the participant was investigated with a CT scanner (Somatom Definition Flash, Siemens Healthineers, Erlangen, Germany). A low-dose CT scan with median effective dose of 2 mSv and slice thickness of 0.6 mm was performed in supine position in full inspiration. No contrast agent was applied. The scan parameters have been reported previously.^17^

### Visual analysis

The study was conducted at six centres in Sweden. The images from each specific CT examination were evaluated by one radiologist. In order to increase the consensus in reporting findings in the eCRF, a 1-day workshop for participating radiologists and a written guide with examples on how to define findings were distributed. A subset of CT scans has been evaluated for interobserver and intraobserver agreement between the participating radiologists and showed good agreement.^17^

Radiologists could review the participants’ previous clinical radiological investigations but had no knowledge of characteristics collected in SCAPIS. Bronchiectasis was defined as bronchial dilatation with respect to the accompanying pulmonary artery. Bronchiectasis, bronchial wall thickening, reticular abnormality, ground-glass opacities, cysts and honeycombing were reported as present or not.^17^

### Clinical and questionnaire data

Participants’ weight and height were measured, and body mass index (BMI) was calculated (kg/m^2^). Forced expiratory volume in 1 s and forced vital capacity were measured with a postbronchodilator spirometry, with postbronchodilator reference values calculated within SCAPIS.^18^ The participants reported their highest education level, their smoking history (categorised as never smoker, ex-smoker or current smoker), and a self-reported current doctor’s diagnosis of asthma. COPD, chronic bronchitis and emphysema were defined as either self-reported condition, or if a diagnosis code for any of these conditions could be identified in previous Swedish healthcare registers (ICD-9, International Classification of Diseases, 9th Revision, diagnosis 491, 492, 496 or ICD-10 diagnosis J41, J42, J43 or J44) at any time prior to study participation.^19^ Airway symptoms were self-reported. Breathlessness was defined as having a modified Medical Research Council score ≥2 (ie, breathless while walking on level ground or worse). Cough was defined as having had dry or productive cough for at least 3 months in a year. Wheezing was defined as any wheezing in the last 12 months.

Participants also reported if they had been diagnosed with inflammatory bowel disease, or a systemic autoimmune disease (including rheumatoid arthritis, Bechterew’s disease, psoriatic arthritis, systemic lupus erythematosus (SLE), Sjögren’s syndrome, vasculitis, systemic sclerosis, sarcoidosis, etc). The following conditions were defined as risk factors for having pulmonary abnormalities: current asthma, ever smoking, inflammatory bowel disease or systemic autoimmune disease.

A subgroup of 8957 participants (30%) gave blood samples for metabolomics analysis, including antacid metabolites (omeprazole, pantoprazole, lansoprazole, ranitidine).

### Statistical analyses

The statistical analysis was performed using STATA/IC V.16.1. For a demographic description of the cohort, we used descriptive statistics (percentages, mean with SD, or median with IQR, as appropriate). We then compared the prevalence of pulmonary abnormalities across nGER groups, using the χ^2^ test. We performed separate multivariate logistic regressions for the different pulmonary abnormalities, with the abnormalities as the outcome and nGER groups as main exposure (‘without nGER’, ‘possible nGER’ and ‘with nGER’, where ‘without nGER’ was the reference group), adjusting for sex (binary), age (continuous), BMI (continuous), smoking history (never smoker, ex-smoker, current smoker), education level (4 categories), current asthma (binary), inflammatory bowel disease (binary) and systemic autoimmune disease (binary). As certain differences exist between the six centres in the reported prevalence of bronchial wall thickening and bronchiectasis, we also adjusted for study centre as a factor variable. Collinearity was assessed by calculating variance inflation factors for the exposures and was below 3 in all cases. Goodness-of-fit was assessed with the Hosmer-Lemeshow χ^2^ test, which found a good model fit. Airway symptoms were not included in the analysis to avoid the risk of collider bias.

To investigate potential confounding by antacid use, we performed the same multivariate analyses on the subgroup with metabolomics data on antacid use, in a complete-case manner.

Multivariate analyses on subgroups were performed to investigate the association between pulmonary abnormalities and nGER among patients with or without other risk factors for pulmonary abnormalities. We performed separate multivariate logistic regressions for the different pulmonary abnormalities, with the abnormalities as the outcome and nGER groups as main exposure, adjusting for sex, age, BMI and education level, and stratified by the presence or absence of risk factors for having pulmonary abnormalities. Risk factors for pulmonary abnormalities were defined as being ex-smoker, current smoker, having current asthma, inflammatory bowel disease or systemic autoimmune disease. Those with any of these risk factors were analysed in the ‘with risk factors’ strata, otherwise they were included in the ‘without risk factors’ strata. We also performed the same logistic regressions with interaction between nGER status and risk factor status. A p<0.05 was considered statistically significant.

### Role of the funding source

The funding sources had no part in the writing of this article or the decision to submit it.

## Results

### Population characteristics

Characteristics for the eligible sample are presented in table 1, divided into three groups: without, possible and with nGER. Participants with nGER had a higher BMI, with a more than two points higher BMI on average compared with those without nGER (median: 28.3 vs 25.9, respectively). Almost 17% of those with nGER were current smokers, compared with 12% of those without nGER. Those with nGER also had a lower education level and had more comorbidities such as asthma, COPD and autoimmune diseases. Lung function was similar among those with and without nGER, but airway symptoms were more common (table 1).

**Table 1:** Participant characteristics stratified by; without, possible or with nGER

	Without nGER	Possible nGER	With nGER
	N=22 184	N=3964	N=2698
Female, n (%)	11 535 (52.0)	1877 (47.4)	1493 (55.3)
Age (years), mean (SD)	57.4 (4.3)	57.7 (4.3)	57.7 (4.4)
BMI (kg/m^2^), median (IQR)	25.9 (23.6–28.8)	27.4 (24.9–30.6)	28.3 (25.4–31.3)
Education level, n (%)			
University	10 406 (47.0)	1619 (41.0)	1016 (37.9)
High school	9843 (44.5)	1897 (48.1)	1332 (49.7)
Elementary school	1768 (8.0)	401 (10.2)	287 (10.7)
Not finished any school	110 (0.5)	30 (0.8)	43 (1.6)
Smoking history, n (%)			
Never smoker	11 519 (52.6)	1787 (45.5)	1196 (44.6)
Ex-smoker	7828 (35.7)	1547 (39.4)	1042 (38.9)
Current smoker	2554 (11.7)	592 (15.1)	442 (16.5)
Current asthma, n (%)	1234 (5.6)	291 (7.5)	253 (9.6)
COPD, chronic bronchitis or emphysema, n (%)	329 (1.5)	77 (1.9)	100 (3.7)
Inflammatory bowel disease, n (%)	235 (1.1)	48 (1.2)	39 (1.5)
Autoimmune disease, n (%)	735 (3.4)	174 (4.5)	151 (5.7)
FEV1, % predicted, mean (SD)	98.1 (13.2)	96.9 (13.2)	96.4 (14.2)
FVC, % predicted, mean (SD)	99.7 (12.4)	98.5 (12.4)	98.3 (12.8)
Wheezing, n (%)	1104 (5.0)	382 (9.8)	398 (15.1)
Breathlessness, n (%)	644 (2.9)	209 (5.4)	311 (11.8)
Dry cough, n (%)	988 (4.5)	248 (6.5)	245 (9.4)
Productive cough, n (%)	1124 (5.2)	336 (8.8)	354 (13.6)

Data are presented as n (%), median (IQR) or mean (SD).

BMI, body mass index; COPD, chronic obstructive pulmonary disease; FEV1, forced expiratory volume in 1 s; FVC, forced vital capacity; nGER, nocturnal gastro-oesophageal reflux.

### Association between nGER and pulmonary abnormalities

Among those with nGER, bronchial wall thickening was more common compared with those without nGER (9.7% vs 7.4%, respectively, p<0.001), and cysts were less common (1.8% vs 2.6%, respectively, p=0.02) (table 2). After adjustment for sex, age, BMI, smoking history, education level, current asthma, inflammatory bowel disease, systemic autoimmune disease and centre, nGER was negatively associated with cysts, with the OR of 0.65 (95% CI 0.48 to 0.89) for cysts (table 3). These results were largely unchanged when also adjusted for the use of antacids, in a subpopulation of 8957 participants (thereof 392 were antacid users, 4.4%) (table 4).

**Table 2:** Pulmonary abnormalities on chest CT for the participants divided into three groups; without, possible and with nocturnal gastro-oesophageal reflux (nGER)

	Without nGER	Possible nGER	P value	With nGER	P value
	N=22 184	N=3964		N=2698	
Bronchial wall thickening, n (%)	1621 (7.4)	345 (8.8)	0.002*	256 (9.7)	<0.001*
Bronchiectasis, n (%)	582 (2.7)	113 (2.9)	0.40	84 (3.2)	0.12
Reticular abnormalities, n (%)	299 (1.4)	63 (1.6)	0.23	47 (1.8)	0.09
Honeycombing, n (%)	40 (0.2)	5 (0.1)	0.45	5 (0.2)	0.94
Cysts, n (%)	561 (2.6)	102 (2.6)	0.86	47 (1.8)	0.02*
Ground glass opacities, n (%)	1439 (6.6)	281 (7.2)	0.15	173 (6.6)	0.97

Univariate analysis on the whole cohort. Data are presented as n (%), statistically significant p values are in bold type. P values calculated with χ2 test, with ‘without nGER’ as reference category.

* Cramér’s V<0.1.

CT, computed tomography; nGER, nocturnal gastroesophageal reflux.

**Table 3:** Relation between nGER and pulmonary abnormalities on chest CT, adjusted for sex, age, BMI, smoking history, education level, current asthma, inflammatory bowel disease, autoimmune disease and study centre

	Possible nGER	P value	With nGER	P value
	N=3964		N=2698	
Bronchial wall thickening	1.03 (0.90 to 1.17)	0.71	1.08 (0.93 to 1.25)	0.33
Bronchiectasis	1.07 (0.86 to 1.31)	0.55	1.20 (0.94 to 1.53)	0.14
Reticular abnormalities	1.08 (0.82 to 1.43)	0.58	1.20 (0.87 to 1.65)	0.28
Honeycombing	0.59 (0.23 to 1.50)	0.27	0.84 (0.32 to 2.15)	0.71
Cysts	0.97 (0.78 to 1.21)	0.82	**0.65 (0.48 to 0.89**)	**0.01**
Ground glass opacities	1.00 (0.87 to 1.15)	0.96	0.89 (0.75 to 1.05)	0.17

Data are presented as OR (95% CI). Statistically significant p values are in bold type. P values calculated with separate logistic regressions, ‘without nGER’ as reference category.

Participants are divided in possible or with nGER.

BMI, body mass index; nGER, nocturnal gastro-oesophageal reflux.

**Table 4:** Relation between nGER and pulmonary abnormalities on chest CT, in a subpopulation with metabolomics data for antacids (n=8957)

	Possible nGER	P value	With nGER	P value
	N=1218		N=822	
Bronchial wall thickening	1.19 (0.94 to 1.51)	0.15	1.06 (0.80 to 1.40)	0.70
Bronchiectasis	0.91 (0.55 to 1.49)	0.70	1.00 (0.56 to 1.78)	0.99
Reticular abnormalities	1.51 (0.67 to 3.40)	0.32	0.60 (0.14 to 2.57)	0.49
Honeycombing	0.30 (0.04 to 2.28)	0.24	0.45 (0.06 to 3.53)	0.45
Cysts	1.26 (0.79 to 2.01)	0.33	**0.45 (0.20 to 0.98**)	**0.046**
Ground glass opacities	1.06 (0.74 to 1.51)	0.76	0.79 (0.51 to 1.24)	0.31

Data are presented as OR (95% CI). Statistically significant p values are in bold type. P values calculated with separate logistic regressions, ‘without nGER’ as reference category.

Analysis adjusted for sex, age, BMI, smoking history, education level, current asthma, inflammatory bowel disease, autoimmune disease, study centre and antacid use. Participants are divided in possible or with nGER.

BMI, body mass index; nGER, nocturnal gastro-oesophageal reflux.

**Figure 1:**
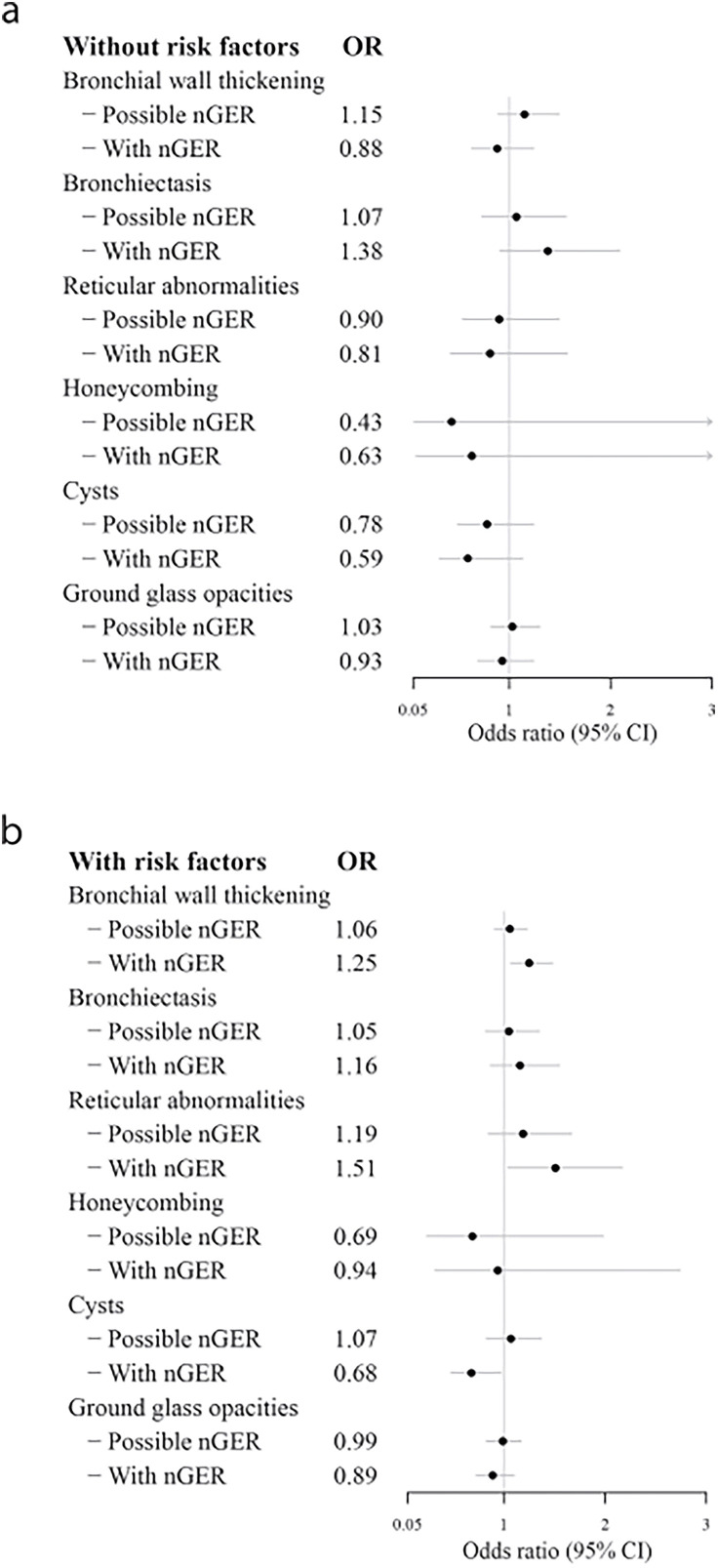
Relation between nGER and pulmonary abnormalities on chest CT scan, adjusted for sex, age, BMI, education level and centre, with ‘without nGER’ as the reference group. The analysis is stratified: (a) only participants without common risk factors for pulmonary abnormalities (never smokers, without asthma, without inflammatory bowel disease and without systemic autoimmune disease, n=2555); (**b**) only participants with any risk factor for pulmonary abnormalities (ex-smokers, current smokers, current asthma, inflammatory bowel disease or systemic autoimmune disease, n=4004). Data are presented as OR (95% CI). The 95% CI for honeycombing in (a) extend beyond the graph’s upper limit: Possible nGER: 0.06–3.28; With nGER: 0.08–4.87. BMI, body mass index; nGER, nocturnal gastro-oesophageal reflux.

However, these associations were not consistent in the group without risk factors for lung abnormalities (never smokers, without asthma, without inflammatory bowel disease and without autoimmune disease, figure 1a). For the group of participants with risk factors for lung abnormalities, bronchial wall thickening (adjusted OR (95% CI): 1.25 (1.07 to 1.48)) and reticular abnormalities (adjusted OR (95% CI): 1.51 (1.04 to 2.17)) were more common, but cysts were significantly less common among those with nGER compared with those without nGER (figure 1b). The associations between nGER and bronchial wall thickening or reticular abnormalities were similar across the different risk factors for pulmonary abnormalities (online supplemental figure S1,S2).

Among participants with nGER, respiratory symptoms were more common among those with bronchial wall thickening or reticular abnormalities, although 49% and 53%, respectively, had no respiratory symptoms in spite of having visible abnormalities on CT (online supplemental table S1,S2).

When the regression models were performed with interaction between nGER and risk factor status (with vs without the risk factors above), no significant interaction was seen.

## Discussion

In this large, multicentre, general population study on middle-aged adults, no significant association was found between nGER and pulmonary abnormalities on chest CT, especially among those without other risk factors for pulmonary abnormalities, regardless of antacid use. On the other hand, among participants with risk factors for having pulmonary abnormalities (smoking history, asthma, inflammatory bowel disease or autoimmune disease), nGER was independently associated with an increased prevalence of reticular abnormalities and bronchial wall thickening, and with a decreased prevalence of cysts. Thus, the hypothesis that nGER is a risk factor for pulmonary abnormalities in the general population could not be supported by our results. Our data suggest that in the general population, nGER should not raise concern for a subsequent development of a serious respiratory disease, unless other risk factors for pulmonary abnormalities are present.

The reason why nGER is associated with pulmonary abnormalities only among those with other risk factors for pulmonary abnormalities is not clear. Possibly, nGER may be a risk factor for pulmonary abnormalities only in the context of other conditions, acting as potentiating factors. For example, smoking decreases protective mechanisms such as cough and reduces basal tone of the lower oesophageal sphincter, thus theoretically increasing the risk for microaspirations.^20 21^ Autoimmune disease could lead to aberrant immune reactions to gastro-oesophageal aspirate, although this has to our knowledge not been studied. Alternatively, nGER may be a comorbidity indicating a more severe disease among those with other risk factors of pulmonary abnormalities. This can be exemplified by systemic sclerosis, where more severe nGER associates with a more severe ILD, but the directionality is not established and could be two independent markers of disease progression.^22 23^ In our cohort, we also found respiratory symptoms to be more common among those with nGER, which may further support that nGER is rather an indicator of more severe disease than a primary risk factor for disease development. Still, lung function was largely similar between those with and without nGER, suggesting that the current findings represent early disease.

The association between nGER and bronchial wall thickening, indicating structural changes in the airways, was intriguing. This supports the hypothesised role for microaspirations in some cases of bronchial disease,^23^ but was only seen among those with other risk factors for pulmonary abnormalities. No significant association was seen with bronchiectasis; however, bronchiectasis was rather uncommon in this cohort. Collectively, our data support a role for nGER in bronchial disease in selected cases.

On the other hand, the general lack of association between nGER and interstitial pulmonary abnormalities is especially interesting in the context of the literature regarding IPF and nGER. IPF is a serious, progressive ILD, where nGER is a common comorbidity and has been hypothesised to cause or worsen the disease.^24 25^ The reported prevalence of nGER in IPF varies greatly, with reported prevalence rates from 0% to 94%.^26^ However, the evidence has been conflicting, and high-quality studies are lacking. Cough, a common symptom of IPF, can cause a rise in the intra-abdominal pressure, and thereby induce reflux.^27^ Microaspiration of gastric contents may indeed cause cellular damage in the lungs and cause exacerbations in more severe IPF, but the extent of this in a clinical context, especially in early IPF disease stages, is debatable.^23 24^ Treatment studies for nGER have also not convincingly shown a benefit in IPF outcomes, but prospective studies are scarce.^24 28^ One possible explanation for the apparent association between IPF and nGER could be reverse causation, that is, that nGER develops in patients with IPF as a part of the disease process. Increased work of breathing and distortion of mediastinal structures in IPF may predispose to increased nGER.^28^ In our cohort, IPF was not specifically defined, making it difficult to draw conclusions regarding IPF. On the other hand, a lack of association with honeycombing, one of the hallmark findings of IPF, as well as reticular abnormalities, indicates a lack of association between nGER and IPF-related CT abnormalities. Therefore, while it is still possible that nGER may have an impact in later stages of IPF, our data do not support that nGER has a role in early development of ILD, including IPF, if no other risk factors are present.

In the present study, nGER was associated with a lower prevalence of cysts, even when adjusted for BMI and age. Cysts have been associated with a lower BMI and higher age.^29^ This finding was unexpected, and the reason is unclear. In part, it could be explained by the low prevalence of cysts among the participants. We, therefore, suggest caution in the interpretation of this result, and further studies are needed to explore this finding.

### Clinical implications

Our findings have several clinical implications. First, in patients with risk factors such as smoking, asthma or autoimmune disease, nGER may predispose to both reticular abnormalities and bronchial wall thickening, suggesting that nGER treatment may be indicated. This is of importance, especially as reticular abnormalities indicate fibrotic lung changes, which may progress to irreversible lung damage.^30^ Bronchial wall thickening suggests airway inflammation and associates with more exacerbations and worse symptom control in asthmatics.^31^ However, the association with bronchial wall thickening was relatively weak, and therefore, nGER should not be overemphasised as other risk factors may be more important. Around half of the patients with nGER and bronchial wall thickening or reticular abnormalities also had respiratory symptoms, indicating that some (although not all) may be captured from reported symptoms. It is therefore not clear if screening with CT should be recommended in this group based on nGER alone, which would require a further analysis including health economics. In the general population, nGER should not raise suspicion of ILD if no other risk factors are present.

### Strengths and limitations

The main strengths of this study are the very large general population sample, with a standardised and detailed evaluation of respiratory health including detailed questionnaires and chest CT scans. The age span of 50–64-year-old adults is representative of a population where early radiological stages of pulmonary abnormalities may be found but also limits the generalisability of the results. Some further limitations exist in this study. First, we did not have detailed information on the use of antacids for the whole cohort, which could potentially diminish the symptoms of nGER. This could potentially lead to a misclassification of some participants with nGER as being without nGER. However, wielding metabolomics data to identify antacid use in a subcohort showed no modification by antacids, even though metabolomics data have general shortcomings—it does not reveal the duration of treatment, for example. Our definition of nGER was only questionnaire-based, which may lead to misclassification. However, in our previous work, a similar definition of nGER was found to be representative and relevant for studying associations to airways diseases.^2^ Second, asthma medications could influence the bronchial wall thickness as either an increase or decrease of the wall thickness depending on how optimised the treatment is and the individual’s compliance to treatment. Our data did unfortunately not permit analysis of such effects. We also lacked data on cancer therapies or radiotherapy with the potential of inducing lung damage, but these therapies are generally rare and are, therefore, unlikely to significantly impact our findings. Data on ethnicity were lacking. However, as the majority of participants were of Scandinavian origin, we suspect that ethnicity would not have impacted our results significantly. Also, our study could not address clinically established ILD owing to their rarity. Indeed, findings of more advanced ILD such as honeycombing were infrequent, but early-stage findings such as reticular abnormalities were more common. Third, as this study was cross-sectional, we could not evaluate any temporal associations.

## Conclusions

In the present study, nGER associated with bronchial wall thickening and reticular abnormalities only among individuals with other risk factors for developing pulmonary abnormalities on chest CT, but not among those without such risk factors. This indicates that, in some cases, nGER may have a role in the development of respiratory disease. In an otherwise healthy general population, nGER should not raise suspicion of pulmonary abnormalities.

## Supplementary material

10.1136/thorax-2024-222570 online supplemental file 1

10.1136/thorax-2024-222570 online supplemental file 2

## Data Availability

Data are available on reasonable request.
