# Supplementary material for: Nocturnal gastro-oesophageal reflux and pulmonary abnormalities on chest CT in a general population: the Swedish CArdioPulmonary BioImage Study
Source: Thorax. 2025 Aug 10;81(1):e222570. doi: 10.1136/thorax-2024-222570 (PMC12772615; doi:10.1136/thorax-2024-222570)
Supplement: online supplemental file 1 [file thorax-81-1-s001.docx]

**Supplementary data**

**Methods**

To evaluate if the association between nGER and bronchial wall thickening or reticular abnormalities were driven by any specific risk factor, we performed separate logistic regressions by each risk factor for pulmonary abnormalities (former / current smoker, current asthma, inflammatory bowel disease, systemic autoimmune disease), with nGER as main exposure and bronchial wall thickening / reticular abnormalities as the outcome.

**Results – Supplementary figures and tables**


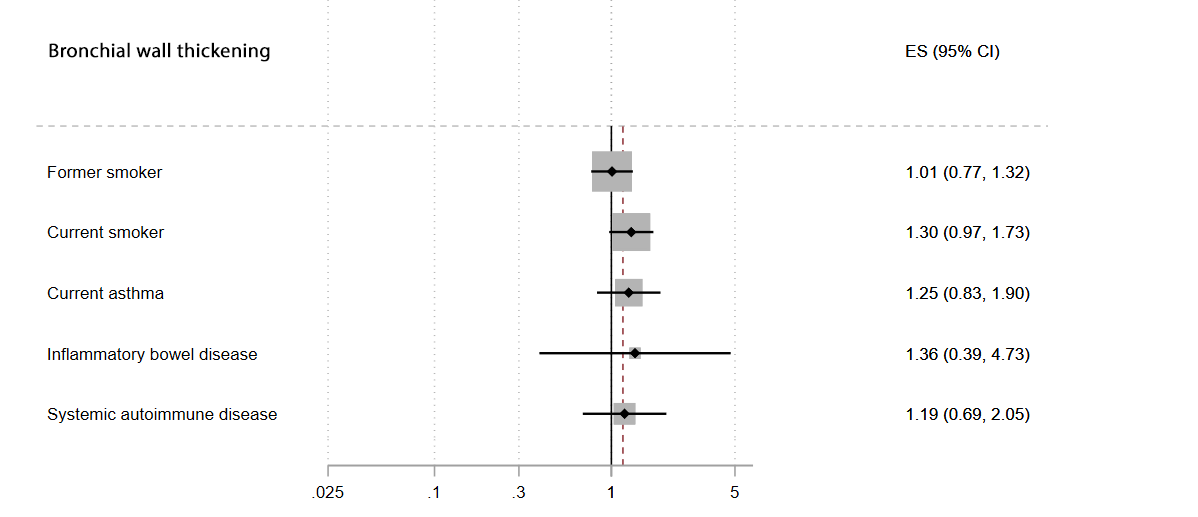


**Figure S1**. Association between bronchial wall thickening and having nGER (‘Without nGER’ as reference category), stratified by identified risk factors for pulmonary abnormalities. Adjusted for age, sex, BMI, education level and study center.

**
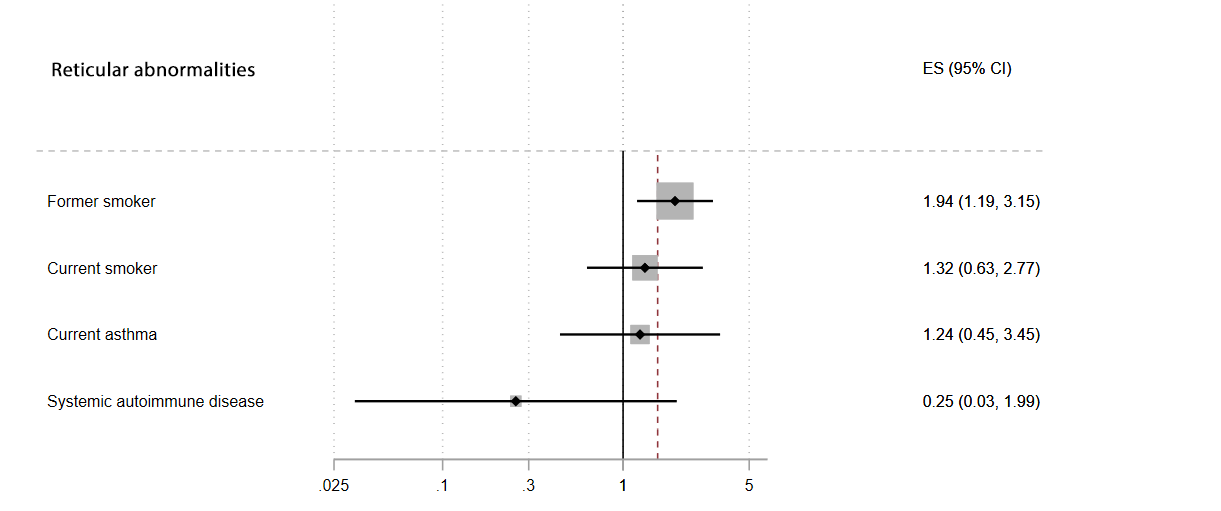
Figure S2**. Association between reticular abnormalities and having nGER (‘Without nGER’ as reference category), stratified by identified risk factors for pulmonary abnormalities. Model did not converge on inflammatory bowel disease, therefore that group was not analyzed. Adjusted for age, sex, BMI, education level and study center.

**Table S1.** Respiratory symptoms among those with nGER, divided by presence or absence of bronchial wall thickening on chest CT scan.

|  | Normal bronchi | Bronchial wall thickening | p-value* |
| --- | --- | --- | --- |
|  | N=2,381 | N=256 |  |
| Wheezing, n (%) | 306 (13.1%) | 80 (32.5%) | <0.001 |
| Breathlessness, n (%) | 255 (11.0%) | 42 (16.7%) | 0.007 |
| Cough, n (%) |  |  | <0.001 |
| Dry cough | 210 (9.2%) | 32 (13.1%) |  |
| Productive cough | 283 (12.4%) | 63 (25.7%) |  |
| Any respiratory symptom, n (%) | 702 (31.7%) | 121 (51.1%) | <0.001 |

*Comparisons evaluated with chi-squared tests.

**Table S2.** Respiratory symptoms among those with nGER, divided by presence or absence of reticular abnormalities on chest CT scan.

|  | No reticular abnormalities | With reticular abnormalities | p-value* |
| --- | --- | --- | --- |
|  | N=2,591 | N=47 |  |
| Wheezing, n (%) | 376 (14.8%) | 10 (21.7%) | 0.19 |
| Breathlessness, n (%) | 290 (11.5%) | 7 (14.9%) | 0.47 |
| Cough, n (%) |  |  | 0.007 |
| Dry cough | 232 (9.3%) | 9 (20.0%) |  |
| Productive cough | 336 (13.5%) | 10 (22.2%) |  |
| Any respiratory symptom, n (%) | 801 (33.2%) | 21 (46.7%) | 0.059 |

*Comparisons evaluated with chi-squared tests.
