# Supplementary material for: Nocturnal gastro-oesophageal reflux and pulmonary abnormalities on chest CT in a general population: the Swedish CArdioPulmonary BioImage Study
Source: Thorax. 2025 Aug 10;81(1):e222570. doi: 10.1136/thorax-2024-222570 (PMC12772615; doi:10.1136/thorax-2024-222570)
Supplement: online supplemental file 2 [file thorax-81-1-s002.pptx]

## Slide 1
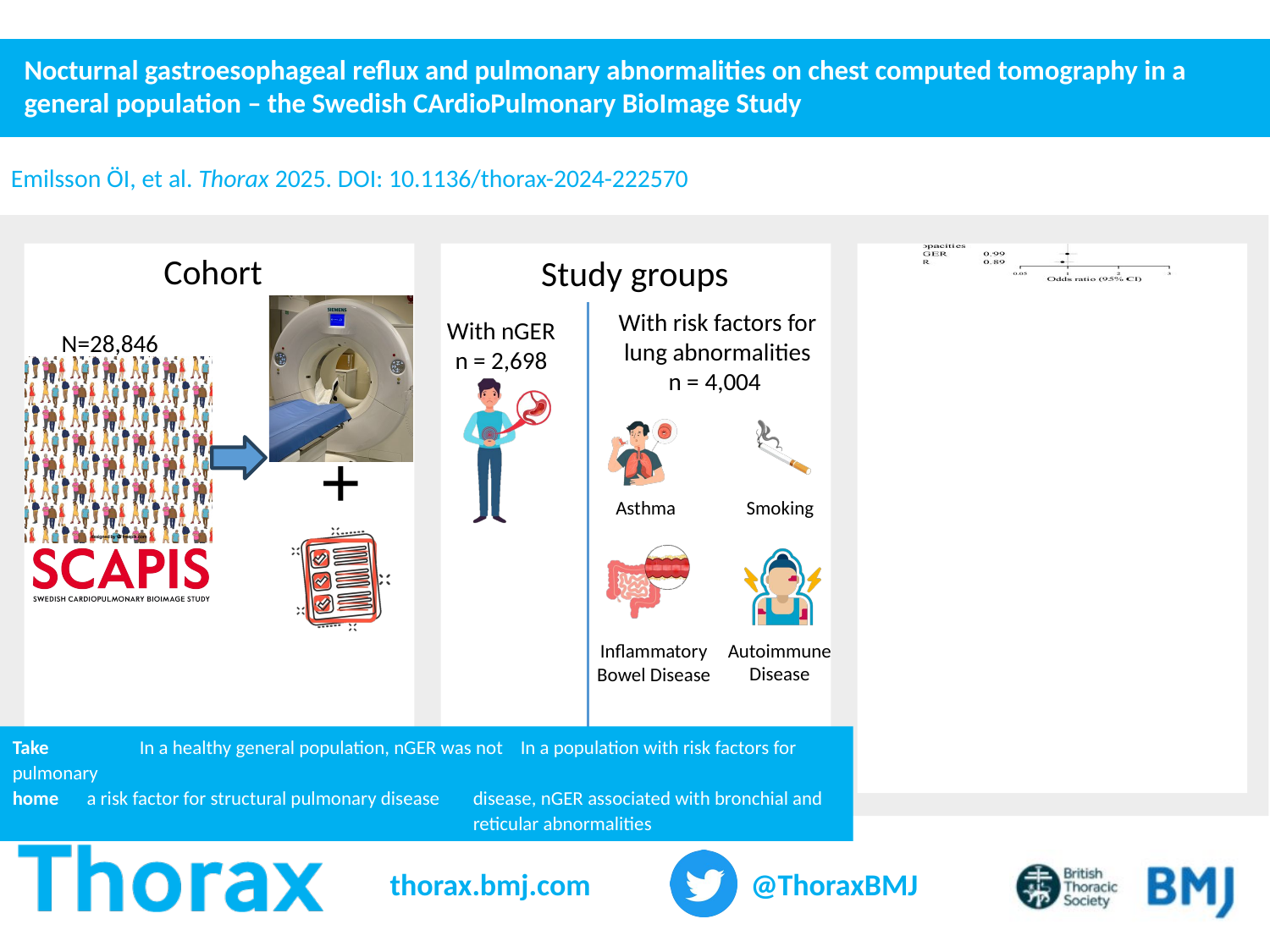

Nocturnal gastroesophageal reflux and pulmonary abnormalities on chest computed tomography in a general population – the Swedish CArdioPulmonary BioImage Study
Emilsson ÖI, et al. Thorax 2025. DOI: 10.1136/thorax-2024-222570
Cohort
Study groups
Manuscript Title
+
With risk factors for lung abnormalities
n = 4,004
With nGER
n = 2,698
N=28,846
Asthma Smoking
Autoimmune Disease
Inflammatory Bowel Disease
© Author(s) (or their employer(s) 2019. Re-use permitted under CC BY. Published by BMJ.
Take	In a healthy general population, nGER was not 	In a population with risk factors for pulmonary
home	a risk factor for structural pulmonary disease	disease, nGER associated with bronchial and
		reticular abnormalities
thorax.bmj.com @ThoraxBMJ
